# Platelet–Leukocyte Aggregate and Interleukin‐6: An Emerging Perspective on a New Diagnostic and Therapeutic Clue for Acute Coronary Syndrome, A Case–Control Study

**DOI:** 10.1002/hsr2.70209

**Published:** 2024-12-11

**Authors:** Mohammad Ghorbani, Davood Bashash, Mohamad Esmail Gheydari, Mohammad Hossein Mohammadi, Hojat Shahraki, Somayeh Yazdanparast, Keyvan Olazadeh, Nazli Atashzar, Mohsen Hamidpour

**Affiliations:** ^1^ Department of Hematology and Blood Banking School of Allied Medical Sciences, Shahid Beheshti University of Medical Sciences Tehran Iran; ^2^ Laboratory Hematology and Transfusion Medicine, Department of Medical Laboratory Sciences Faculty of Allied Medicine, Gonabad University of Medical Sciences Gonabad Iran; ^3^ Department of Cardiology Taleqani Hospital, Shahid Beheshti University of Medical Sciences Tehran Iran; ^4^ HSC Research Center ‐ Department of Hematology and Blood Banking School of Allied Medical Sciences, Shahid Beheshti University of Medical Sciences Tehran Iran; ^5^ Department of Heamatology, Faculty of Medical Sciences Tarbiat Modares University Teharn Iran; ^6^ Research Center for Social Determinants of Health, Research Institute for Endocrine Sciences Shahid Beheshti University of Medical Sciences Tehran Iran; ^7^ Department of Biostatistics School of Allied Medical Sciences, Shahid Beheshti University of Medical Sciences Tehran Iran

**Keywords:** acute coronary syndrome, atherosclerosis, cytokine, platelet‐monocyte aggregate, platelet‐neutrophil aggregate

## Abstract

**Background and Aims:**

Acute coronary syndrome (ACS) is one of the most important cardiovascular diseases. The rupture of atherosclerotic plaques in coronary arteries is considered the underlying pathophysiology of ACS. The interaction between cytokines and leukocytes in the presence of platelets results in platelet–leukocyte aggregate (PLA). Monocytes, neutrophils, and cytokines are prime factors that promote PLA formation, which leads to atherosclerotic plaque progression and subsequent ACS development. This study aimed to investigate PLA (PMA and PNA) formation and cytokine (IL‐6 and TNF‐α) levels as well as the correlation between them in ACS patient samples to identify diagnostic markers.

**Methods:**

A total of 30 patients with ACS and 24 healthy controls participated in this study. Flow cytometry analysis was performed to evaluate PLA formation, and the serum levels of cytokines were assessed by ELISA. The Pearson's correlation coefficient and ROC curve were calculated to investigate the correlation between the parameters and their diagnostic value, respectively.

**Results:**

The results showed that PMA, PNA, and IL‐6 levels were significantly higher in ACS patients than in healthy controls. Additionally, TNF‐α levels were not significantly increased in the patient group. In addition, the Pearson's correlation coefficient results revealed a direct linear and statistically significant relationship between PMA‐IL‐6 and PNA‐IL‐6 as well as a direct linear but statistically nonsignificant relationship between IL‐6‐TNF‐α and PMA–PNA, whereas a convers linear but nonsignificant correlation was shown between PMA and TNF‐α and no correlation was detected between PNA and TNF‐α. Finally, ROC curve analysis revealed that the PMA, PNA, and IL‐6 can have diagnostic value.

**Conclusion:**

In conclusion, the PMA, PNA, and IL‐6 can be used as powerful diagnostic markers in ACS patients. Therefore, disrupting PMA and PNA formation and inhibiting cytokine production may be new strategies for the treatment of ACS. However, further investigations are required to explore these parameters in the clinical diagnosis of ACS.

## Summary

- PLA and cytokines play a critical role in ACS pathogenesis.
- PMA and PNA have diagnostic value in ACS with high sensitivity and specificity.
- Cytokines, especially IL‐6 could be a powerful diagnostic marker in ACS.
- Inhibiting PLA formation and cytokines secretion provides a therapeutic clue for ACS.

**Abbreviations**
ASC: acute coronary syndrome
CAA: coronary artery abnormalities
CAD: coronary artery disease
CCS: chronic coronary syndrome
CVD: cardiovascular disease
IL‐6: interleukin‐6
MI: myocardial ischemia
PLA: platelet–leukocyte aggregate
PMA: platelet–monocyte aggregate
PNA: platelet–neutrophil aggregate
TNF‐α: tumor necrosis factor α

## 1 Introduction

Acute coronary syndrome (ACS) is a major cause of mortality and morbidity that affects millions of people worldwide [1, 2]. Following the rupture of atherosclerotic plaques, platelets activate, and initiate thrombus formation inside coronary arteries, which can obstruct blood flow and cause myocardial ischemia (MI). Inflammation plays a pivotal role in the pathogenesis of atherosclerosis, and its complications are particularly correlated with the development of ACS [3, 4, 5]. An increasing amount of evidence indicates that inflammatory activity has a detrimental role in the pathophysiology of ACS, initiating and advancing atherosclerosis, promoting destabilization and deterioration of plaques, and reacting to myocardial necrosis [6].

The biochemical and cellular phases can be considered in the process of atherosclerosis [7]. Two main cell types that contribute to the pathogenesis of atherosclerosis and atherothrombosis are platelets and leukocytes, which are implicated in the incidence of cardiovascular diseases (CVDs), particularly ACS [7, 8, 9, 10]. Platelet–leukocyte aggregate (PLA) formation represents one of the most critical interactions between platelets and leukocytes. PLA is characterized by heterotypic combinations comprising at least one platelet and one leukocyte [11].

PLA includes various subtypes of leukocytes, but two main subtypes include platelet–monocyte aggregate (PMA) and platelet–neutrophil aggregate (PNA) [11]. In healthy individuals, platelets primarily form aggregates with monocytes (4.1%–7.2%), followed by neutrophils (3.7%–5.7%). However, reference ranges are not established due to the absence of a validated protocol for assessing PLA concentrations [11, 12, 13]. However, in individuals with cardiovascular risk factors and acute or stable coronary syndromes, there is an increase in the PLA, suggesting its potential utility as a diagnostic marker [11].

The significant increase in the number of circulating PLAs in patients with atherosclerosis‐related complications has garnered considerable interest. The connection of platelets to leukocytes, in addition to the recruitment of leukocytes to atherosclerotic plaques, causes morphological, functional, and content changes in leukocytes, which lead to the development of atherosclerotic plaques [14].

The dynamics, diversity, and functions of PLAs have been investigated across various cardiovascular conditions, including ACS, chronic coronary syndrome (CCS), peripheral artery disease, heart failure, carotid artery stenosis, and cerebral ischemia [11, 15]. The potential of the PLA as a biomarker extends to diagnosing coronary syndromes, predicting outcomes following revascularization procedures, and monitoring the effectiveness of antiplatelet therapy [11]. Although further research is required to fully elucidate the mechanisms underlying PLAs in atherosclerosis and atherothrombosis, leveraging PLA as an indicator of platelet and/or leukocyte activation and for assessing the risk of atherothrombosis development shows considerable potential [11, 16]. As numerous small‐scale human studies have indicated a potential advantage in utilizing commercially available antiplatelet medications to diminish platelet–leukocyte interactions, coupled with the gradual unraveling of the role of PLAs in atherosclerosis development and atherothrombosis, targeting the PLA therapeutically has emerged as an intriguing prospect for mitigating atherosclerosis progression and atherothrombosis [17].

Studies have shown a significant increase in PLAs in various diseases in addition to CVDs such as type I diabetes mellitus, inflammatory bowel disease, COVID‐19, and Kawasaki disease [18, 19, 20, 21]. This increase in the PLA can be associated with thrombotic events in these diseases [20, 21].

Numerous studies have consistently demonstrated a correlation between CVD and heightened levels of inflammatory biomarkers, such as cytokines. These cytokines, which are released by activated pro‐inflammatory cells, promote the thickening or rupture of atherosclerotic plaques and arterial thrombosis. Specifically, interleukin‐6 (IL‐6), a prominent pro‐inflammatory cytokine, is recognized as a primary instigator of plaque destabilization, atheroprogression, and the generation of high‐sensitivity C‐reactive protein (hs‐CRP), thereby fostering the onset and progression of clinical atherosclerosis. Moreover, IL‐6 levels have been linked to plaque burden, as delineated by intracoronary imaging [6, 22].

Tumor necrosis factor α (TNF‐α), another important cytokine in atherosclerosis, plays an important and key role in the occurrence and development of atherosclerotic plaques. In fact, this cytokine, which has multiple functions, such as inducing vascular adhesion molecules and recruiting white blood cells, especially monocytes and macrophages, as well as suppressing the activation of 7α‐hydroxylase and lipoprotein lipase to stimulate the production of triglycerides in the liver, plays an important and essential role in the development of atherosclerotic plaques, followed by ACS; thus, in new studies, blocking TNF‐α is considered one of the therapeutic goals for ACS [23].

Considering the role of both PLA (PMA and PNA) and cytokine factors (IL‐6 and TNF‐α) in the pathophysiology of ACS, we conducted this prospective study to evaluate PLA and cytokine levels in ACS patients and the associations between PLA and cytokine levels in these patients.

## 2 Materials and Methods

### 2.1 Study Design

This study, as a case–control study, was conducted at Taleqani Hospital in Tehran, Iran, between August 13, 2023, and January 9, 2024, with a focus on individuals diagnosed with ACS. The study received approval from the Ethical Committee of Shahid Beheshti University of Medical Sciences (IR.SBMU.RETECH.REC.1401.65), and informed consent was obtained from all participants in accordance with the Declaration of Helsinki.

### 2.2 Population Study

A total of 54 individuals participated in the study, comprising 30 patients with ACS who visited Taleghani Hospital and 24 healthy volunteers who were under study in 2023. All patients presenting to Taleghani Hospital's emergency department with resting chest pain and ischemic ECG changes (Braunwald Class IIIb) or infarction (with or without elevated troponin) were included in the clinical study. Patients with a history of chest pain or myocardial infarction within 3 months, coronary artery bypass grafting or percutaneous coronary intervention within 6 months, or use of antiplatelet medications other than aspirin were excluded. Enrolled patients were hospitalized prior to angiography. Whole blood samples were collected upon admission for the measurement of PMAs, PNAs, and other circulating biomarkers. Citrated blood and clot samples were obtained before angiography and antiplatelet therapy initiation for PLA and cytokine analysis, respectively. Serum samples were stored at −70°C until testing. Patients with renal, hepatic, inflammatory, hematological, or autoimmune diseases, or those receiving immunosuppressive agents, smoking, a platelet count < 100 × 10^9^/L or > 450 × 10^9^/L, pregnancy, anemia, cancer, infectious disease, any bleeding disorder, and high consumption of antioxidant supplements in the last month were also excluded. Patients with a stenosis of at least 70% in one or more major vessels, as determined by angiography, were considered to have ACS and included in the study. The control group consisted of 24 healthy volunteers matched for age and sex. The exclusion criteria for healthy volunteers were dissatisfaction; a history of any CVD; a platelet count < 100 × 10^9^/L or > 450 × 10^9^/L; anemia; cancer; inflammatory disease; smoking, and the use of any drugs affecting platelet function, such as nonsteroidal anti‐inflammatory drugs (NSAIDs), 1 week before sampling.

### 2.3 Blood Sample Collection

For both patients and the control group, 2 mL of whole blood was collected into a 3.2% (0.105 mol/L) sodium citrate tube for measurement of the PMA and PNA. Additionally, 4 mL was transferred to a clot tube using a 19‐gauge needle to measure the levels of cytokines (IL‐6 and TNF‐α). Additionally, to avoid turbulent flow and cell activation, sampling was performed via direct venipuncture rather than through a cannula (for patients), and the tourniquet was slowly closed and slowly opened and was closed in minimum time. Overall, efforts were made to ensure a smooth blood collection process without vascular stasis, and the samples were promptly transported to the laboratory for marker assessment.

### 2.4 Flow Cytometry Analysis

To detect the PMA and PNA, we utilized the following monoclonal antibodies: isotype IgG1 mouse antihuman CD41‐PE, isotype IgG1 mouse antihuman CD14‐FITC, isotype IgG1 mouse antihuman CD61‐FITC, and isotype IgG1 mouse antihuman CD16‐PE (Beckman Coulter).

To detect PLA, 50 µL of the blood sample was diluted with 450 µL of PBS/EDTA/albumin buffer. One hundred microliters of the diluted whole blood were incubated at room temperature with 10 µL of PE‐conjugated anti‐CD41, FITC‐conjugated anti‐CD14 in a test tube for measurement of PMA, and FITC‐conjugated anti‐CD61, PE‐conjugated anti‐CD16 in another test tube for measurement of PNA. In fact, the markers were added to the wells. Then, the samples were fixed with 500 µL of 1% paraformaldehyde in distilled water. Red blood cell lysis and washing were performed. The cells were resuspended in PBS and analyzed by FACSCalibur flow cytometry with CellQuest software (Becton Dickinson Biosciences). For each sample, an isotype‐matched negative control, antihuman IgG, was used. Subpopulations of leukocytes were identified using a scatter plot of forward verus side scatter. CD14 was utilized to validate the monocyte gate, while CD16 was employed to validate the neutrophil gate. Then, the expression of CD41 on monocytes as a PMA and the expression of CD61 on neutrophil populations as a PNA were detected. The levels of PMA and neutrophil–platelet aggregates were detected as percentages of total monocytes and neutrophils, respectively.

### 2.5 Cytokine Assay

For cytokine assessment (TNF‐α and IL‐6), 5 mL of blood was drawn into a clot tube and centrifuged at room temperature, followed by separation and freezing at −70°C for subsequent analysis. TNF‐α concentrations were determined by employing a commercially available enzyme‐linked immunosorbent assay (ELISA) (DiaMetra kit, Siemens BEP 2000 system). All procedures were executed based on the manufacturer's protocol and within a closed system. IL‐6 concentrations were assessed using a chemiluminescence immunoassay (Siemens kit, IMMULITE 2000 immunoassay system) following the manufacturer's instructions and within a closed system.

### 2.6 Statistical Analysis

Statistical analysis was conducted utilizing the Statistical Package for Social Sciences, version 16.0 (SPSS Inc.). The results are presented as the mean (standard deviation). Significant differences between groups were assessed using the *χ* ^2^ test for categorical variables and Student's *t*‐test for continuous variables. The Pearson's correlation coefficient was employed to explore associations between variables. A *p* < 0.05 was considered significant. ROC curves were generated to investigate whether the PMA, PNA, IL‐6, and TNF‐α indices can serve as suitable markers for distinguishing patients from healthy individuals.

## 3 Results

### 3.1 Basic Characteristics of the Study Population

The baseline laboratory and clinical characteristics of the healthy controls and ACS patients are summarized in Table 1. The average age in the healthy control group was 53.8 years, while in the patient group, it was 57.2 years. Independent Student's *t*‐tests were used to compare the average laboratory indicators between the two groups. The WBC, RBC, HGB, HCT, RDW, FBS, A1C, ALT, AST, and neutrophil counts were significantly different between the healthy control and patient groups (*p* < 0.05). The average RBC, HGB, and HCT were higher in the healthy control group than in the patient group, while the average WBC, RDW, FBS, A1C, ALT, and AST were higher in the patient group than in the healthy control group. Other parameters, including the MCV, MCH, MCHC, PLT, lymphocyte count, and monocyte count, did not significantly differ between the two groups of patients and controls (*p* > 0.05).

**Table 1:** Demographic and clinical characteristics of healthy controls and ACS patients.

Parameter		Healthy control (*n* = 24)	ACS patients (*n* = 30)	*p* value
Age		53.8 (5.14)	57.2 (8.62)	0.125
Sex	Male	15 (62.5)	18 (60)	0.851
	Female	9 (37.5)	12 (40)	
Hematology	WBC	7629.7 (2064.3)	9206.1 (1998/09)	0.008*
	RBC	4,868,452.71 (43822.32)	4,372,421.48 (767,575.20)	0.004*
	HGB	13.28 (2.01)	12.70 (1.56)	0.018*
	HCT	40.12 (4.61)	38.17 (4.12)	0.019*
	MCV	86.88 (2.98)	86.07 (12.51)	0.484
	MCH	28.18 (1.85)	28.82 (2.22)	0.321
	MCHC	34.29 (0.97)	34.08 (1.07)	0.060
	PLT	232,352 (49,998.71)	216,878.22 (53,632.72)	0.267
	RDW	12.19 (2.07)	14.87 (2.01)	0.008*
	Neutrophil	3517.92 (1421.86)	5298 (2018.71)	< 0.001*
	Lymphocyte	2462.85 (898.14)	2218.28 (821.62)	0.201
	Monocyte	528.89 (201.08)	431.17 (108.20)	0.200
Biochemistry	FBS	90.81 (7.32)	128.52 (47.50)	< 0.001*
	A1C	5.89 (1.02)	6.82 (1.56)	< 0.001*
	ALT	21.27 (7.81)	28.75 (11.44)	0.002*
	AST	23.89 (5.61)	29.17 (12.81)	0.031*
	Chol	185.25 (45.6)	132.4 (35.2)	< 0.001
	TG	144.8 (73.1)	109.7 (64.4)	< 0.001
	LDL	111.7 (20.9)	74.5 (18.5)	< 0.001
	HDL	39.8 (6.9)	40.3 (6.2)	0.926

*Note:* Categorical and continuous variables are expressed as the mean (SD) and number (%), respectively. The *p* value was assessed using *χ* ^2^ tests for categorical variables and the *t*‐test for continuous variables.

Abbreviations: A1C, hemoglobin A1C; ALT, alanine aminotransferase; AST, aspartate aminotransferase; Chol, cholesterol; FBS, fasting blood sugar; HCT, hematocrit; HDL, high‐density lipoprotein. HGB, hemoglobin; LDL, low‐density lipoprotein; MCH, mean cell hemoglobin; MCHC, mean corpuscular hemoglobin concentration; MCV, mean cell volume; PLT, platelet; RBC, red blood cell; RDW, red cell distribution width; TG, triglyceride; WBC, white blood cell.

* *p* < 0.05.

### 3.2 Increased PMA and PNA Formation

**Figure 1:**
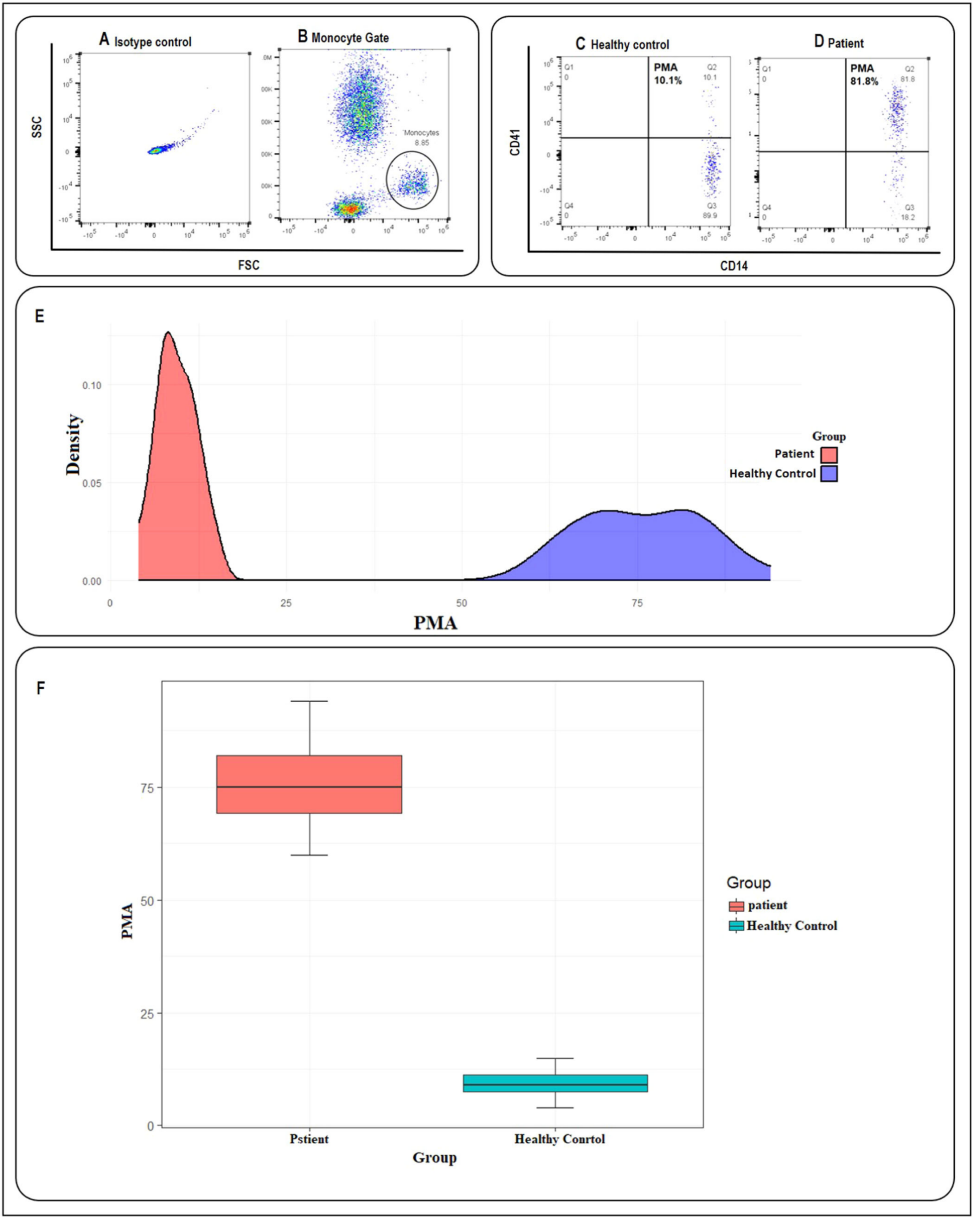
The platelet–monocyte aggregates (PMA) formation in healthy control and acute coronary syndromes (ACS) patients. (A) Isotype control. (B) Monocytes were initially gated based on characteristic forward scatter (FSC) and side scatter (SSC), as indicated within the circle. The population of cells co‐expressing CD14 and CD41 markers was identified as PMA, as illustrated in (C and D). (C) An example of the amount of PMA in a healthy control. (D) An example of the amount of PMA in an ACS patient. (E) The density plot showing the effectiveness of the parameters in distinguishing between patient and healthy groups, the red curve for the patient group and a blue curve for the healthy control group. (F) The box plot showing the comparison of PMA averages in patients (red box) and healthy volunteers (blue box) (75.78 ± 8.74 in patients vs. 9.35 ± 2.8 in controls, *p* < 0.001).

**Figure 2:**
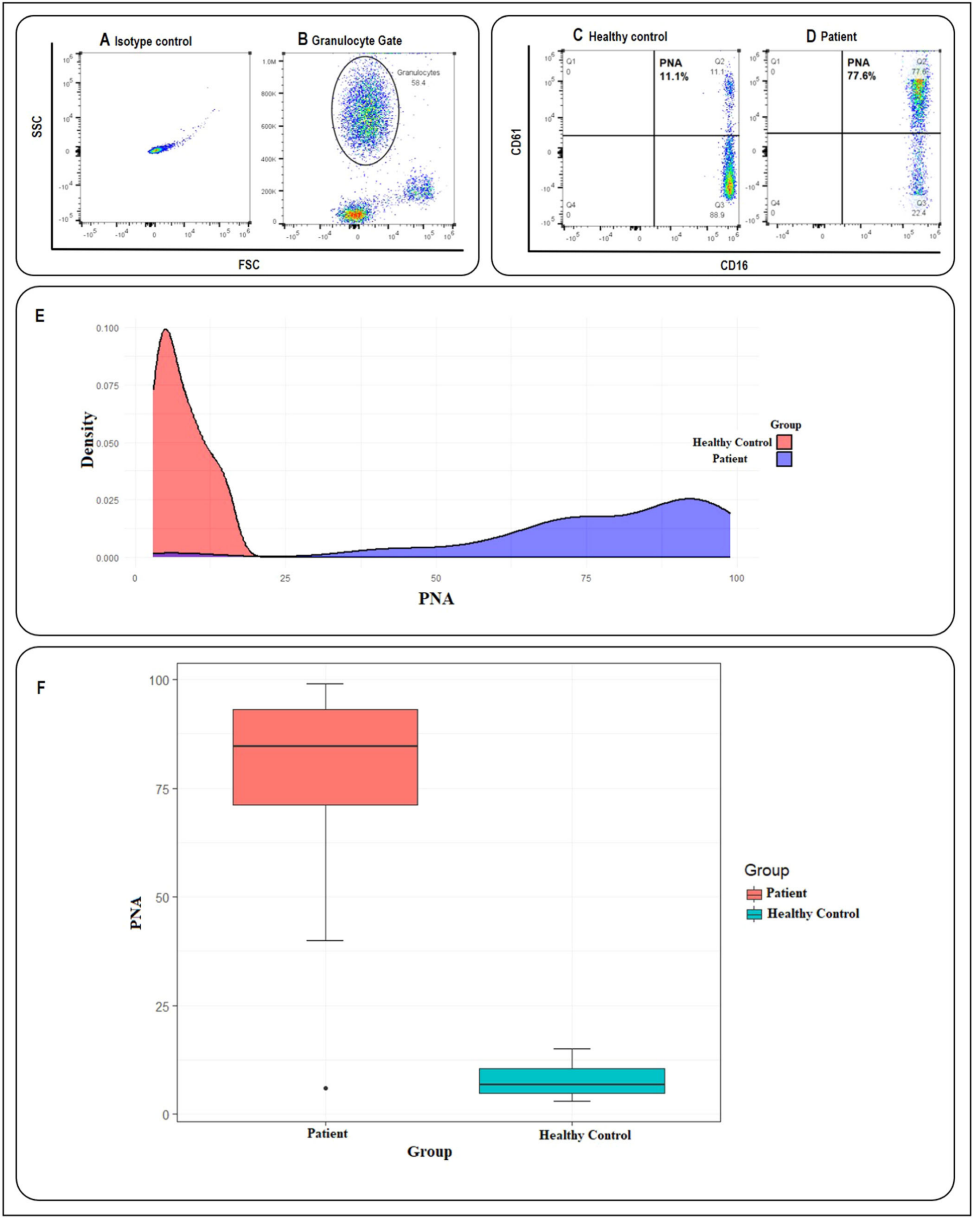
The platelet–neutrophil aggregates (PNA) formation in healthy control and acute coronary syndromes (ACS) patients. (A) Isotype control. (B) Neutrophils were initially gated based on characteristic forward scatter (FSC) and side scatter (SSC), as indicated within the circle. The population of cells co‐expressing CD16 and CD61 markers was identified as PNA, as illustrated in (C and D). (C) An example of the amount of PNA in a healthy control. (D) An example of the amount of PNA in an ACS patient. (E) The density plot, showing the effectiveness of the parameters in distinguishing between patient and healthy groups, the red curve for the patient group and a blue curve for the healthy control group. (F) The box plot showing the comparison of PNA averages in the patient (red box) and healthy volunteers (blue box) (77.88 ± 20.78 in patients vs. 7.82 ± 3.94 in controls, *p* < 0.001).

Flow cytometry analysis was implemented to evaluate PMA and PNA formation. First, we examined the value of the PMA in both the patient and healthy control groups. As illustrated in Figure 1 and Table 2, the results revealed a significant difference in PMA formation between these two groups, and the percentage of PMA in the patient group was significantly higher than that in the control group (75.78 ± 8.74 in patients vs. 9.35 ± 2.80 in healthy controls, *p* < 0.001). Then, the PNA percentage was measured in both the patient and healthy control groups. PNA values were significantly higher in the patient group than in the healthy control group (77.88 ± 20.78 in patients vs. 7.82 ± 3.94 in healthy controls, *p* < 0.001) (Figure 2 and Table 2).

**Table 2:** Comparison of average PMA, PNA, IL‐6, and TNF‐α parameters between healthy patients and patient participants.

Variables	Healthy control (*n* = 24)	ACS patients (*n* = 30)	*p* value
PMA	9.35 (2.80)	75.78 (8.74)	< 0.001
PNA	7.82 (3.94)	77.88 (20.78)	< 0.001
IL‐6	5.04 (1.35)	23.52 (17.13)	< 0.001
TNF‐α	8.60 (2.07)	10.69 (4.77)	0.051

*Note:* Categorical and continuous variables are expressed as the mean (SD) and number (%), respectively. The *p* value was assessed using *χ* ^2^ tests for categorical variables and the *t*‐test for continuous variables.

Abbreviations: ACS; acute coronary syndromes, PMA; platelet–monocyte aggregates, PNA; platelet–neutrophil aggregates, IL‐6; interleukin‐6, TNF‐α; tumor necrosis factor α.

### 3.3 Increased IL‐6 and TNF‐α Plasma Levels in Patients With ACS

**Figure 3:**
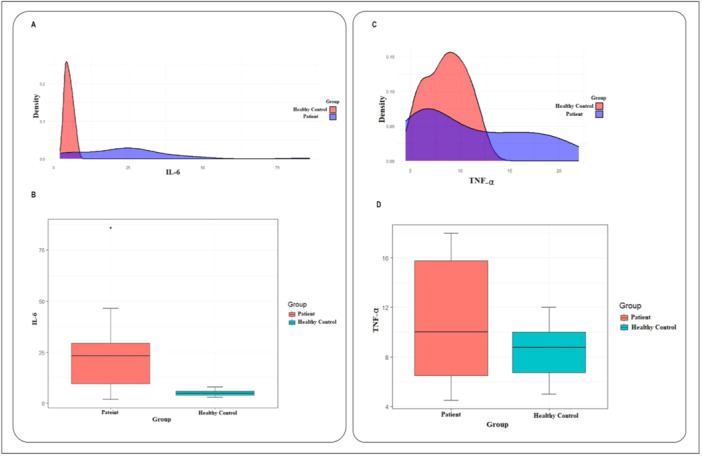
The interleukin‐6 (IL‐6) and tumor necrosis factor α (TNF‐α) plasma levels in healthy control and acute coronary syndromes (ACS) patients. (A) The density plot for IL‐6 in the patient group (blue curve) and healthy control (red curve). (B) The box plot showing the comparison of IL‐6 averages in patients (red box) and healthy control (blue box) (23.52 ± 17.13 in patients vs. 5.04 ± 1.35 in controls, *p* < 0.001). (C) The density plot for TNF‐α in the patient group (blue curve) and healthy control (red curve). (D) The box plot showing the comparison of TNF‐α average in patients (red box) and healthy control (blue box) (10.69 ± 4.77 in patients vs. 8.60 ± 2.07 in controls, *p* > 0.05).

The plasma IL‐6 and TNF‐α levels were examined via chemiluminescence immunoassay and ELISA, respectively. The findings revealed that the level of IL‐6 was markedly different between the two groups and increased significantly in the patient group compared to the healthy control group. As shown in Figure 3A,B and Table 2, the IL‐6 level in the patient group was almost five times higher than that in the healthy control group (23.52 ± 17.13 in patients vs. 5.04 ± 1.35 in controls, *p* < 0.001). Additionally, according to the results shown in Figure 3C,D and Table 2, although higher levels of TNF‐α were detected in the patient group than in the control group, there was no significant difference between the two groups (*p* > 0.05).

### 3.4 Comparisons of PMA, PNA, IL‐6, and TNF‐α Between Healthy Controls and ACS Patients

Independent *t*‐tests were conducted to compare the mean levels of PMA, PNA, IL‐6, and TNF‐α between the patient and control groups. The results are summarized in Table 2. The results revealed that the average PMA index was significantly higher in the patient group (75.78) than in the healthy group (9.35) (*p* < 0.001). Similarly, the average PNA index was significantly higher in the patient group (77.88) than in the control group (7.82) (*p* < 0.001). The average IL‐6 index in the patient group (23.52) was also significantly higher than that in the healthy group (5.04) (*p* < 0.001). Finally, the average TNF‐α index was 10.69 ± 4.77 in the patient group and 8.60 ± 2.07 in the healthy group, which were not significantly different (*p* > 0.05).

### 3.5 Correlations Between PMA, PNA, IL‐6, and TNF‐α in Patients With ACS

**Figure 4:**
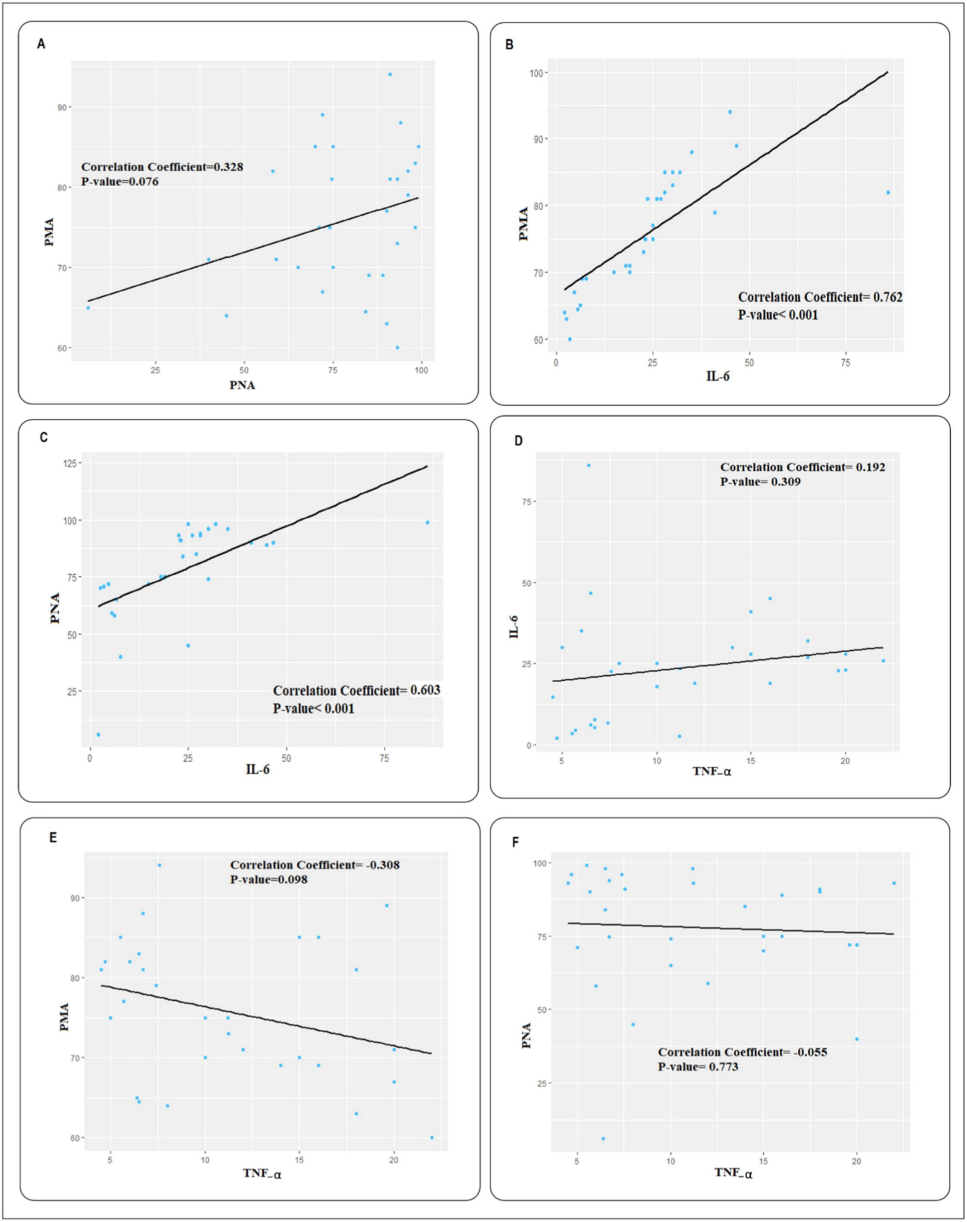
The correlation between platelet–monocyte aggregates (PMA), interleukin‐6 (IL‐6), and tumor necrosis factor α (TNF‐α) in acute coronary syndromes (ACS) patients. (A) The Pearson's correlation coefficient indicates a direct linear but nonsignificant relationship between the PMA and PNA. (B) The Pearson's correlation coefficient indicates a significant and direct linear relationship between the PMA and IL‐6. (C) The Pearson's correlation coefficient indicates a significant and direct linear relationship between the PNA and IL‐6. (D) The Pearson's correlation coefficient indicates a direct linear but nonsignificant relationship between TNF‐α and IL‐6. (E) The Pearson correlation coefficient indicates a reverse linear but nonsignificant relationship between PMA and TNF‐α. (F) The Pearson's correlation coefficient indicates there is no linear relationship between PNA and TNF‐α.

The Pearson's correlation coefficient test was used to assess the linear relationship between the PMA, PNA, IL‐6, and TNF‐α parameters in the patient group. Figure 4 displays a dot chart illustrating the linear relationship between each of the parameters. The analysis revealed a direct linear but nonsignificant statistical relationship between PMA and PNA (*p* > 0.05, Figure 4A), a direct linear and statistically significant relationship between PMA and IL‐6 (*p* < 0.001, Figure 4B) and between PNA and IL‐6 (*p* < 0.001, Figure 4C). Moreover, a direct but nonsignificant relationship was observed between IL‐6 and TNF‐α pentameters (*p* > 0.05, Figure 4D). Additionally, our analysis indicated a reverse linear but nonsignificant relationship between PMA and TNF‐α (Figure 4E) but no linear relationship between PNA and TNF‐α (Figure 4F).

### 3.6 Receiver Operating Characteristic (ROC) Curve and Diagnostic Value of PMA, PNA, IL‐6, and TNF‐α

**Figure 5:**
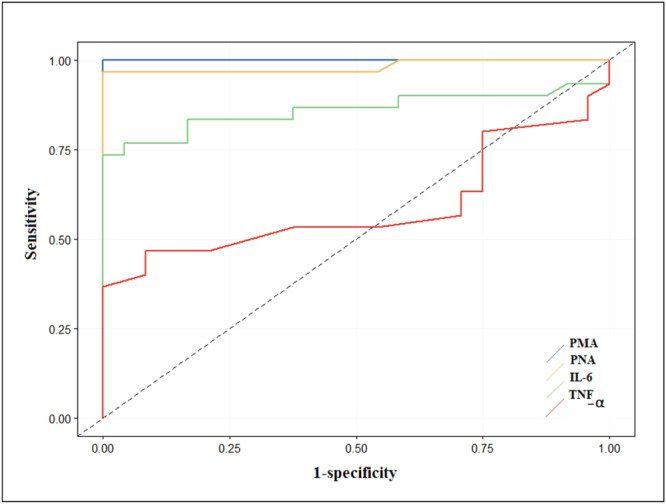
Receiver operating characteristic (ROC) curve analysis of PMA, platelet–neutrophil aggregates (PNA), IL‐6, and TNF‐α in acute coronary syndromes (ACS) patients. The receiver operating characteristic (ROC) curve shows that PMA with the area under the curve (AUC) = 1 (blue line) and PNA with an AUC = 0.982 (yellow line) and IL‐6 with AUC = 0.859 (green line) together are reliable and powerful markers in differentiating the group of patients with ACS and healthy controls, and TNF‐α with AUC = 0.588 (red line) is less effective in differentiating between the two groups.

ROC curve analysis was performed to evaluate the diagnostic performance of PMA, PNA, IL‐6, and TNF‐α. As shown in Figure 5, the area under the curve (AUC) was 1 for PMA (blue line, Figure 5), 0.982 for the PNA variable (yellow line, Figure 5), 0.859 for the IL‐6 variable (green line, Figure 5), and 0.588 for the TNF variable (red line, Figure 5). Therefore, our findings indicated that PMA, PNA, and IL‐6 are the most useful diagnostic markers for ACS, while TNF‐α shows less diagnostic value for distinguishing between healthy controls and ACS patients.

## 4 Discussion

In this study, we investigated two parameters, PLAs and cytokines, in patients with ACS and compared these factors between ACS patients and healthy controls. We aimed to elucidate the relationships between two factors and the pathogenesis and diagnosis of ACS. Notably, PMA and PNA were explored as two subsets of PLA.

Our findings indicate a significant increase in PMAs and PNAs in ACS patients compared with healthy controls. Consistent with these findings, previous studies have reported increased PLA levels in individuals with CVD, especially coronary artery disease (CAD) [11, 21].

The recruitment of neutrophils facilitated by platelets further exacerbates the development of intimal hyperplasia at the site of vascular injury. Additionally, PNA contribute to clot formation by producing procoagulant TFs. This factor interacts with both monocytes and neutrophils, establishing a connection that enhances the process of clot formation and poses additional risks to vascular health [14, 21, 24].

Additionally, PNA play a pivotal role in initiating their respective activations, leading to the generation of thromboxane A2 (TXA2) and other inflammatory factors. These processes give rise to a diverse array of cellular responses, encompassing integrin activation, neutrophil recruitment, platelet aggregation, increased vascular permeability, and potential involvement in vascular injury [21, 24].

In a study conducted by Uneo and colleagues, it was observed that the rate of PNA was significantly higher in patients with Kawasaki disease (KD) compared to those with bacterial infections and normal volunteers. Moreover, within the KD patient group, those with coronary artery abnormalities (CAA) exhibited a notably higher rate of PNAs than those without CAA. This finding suggests that PNA may contribute to thrombosis and prolonged inflammation, thereby increasing the severity of coronary outcomes. These aggregates seem to play a significant role in the pathological developments associated with CAA in KD [21].

In the study of Ott and colleagues, patients with unstable angina showed a significant increase in neutrophil–platelet adhesion compared with patients with stable angina. It thereby reveals that neutrophil–platelet adhesion contributes to the activation of inflammatory cells and presents a novel link between thrombotic processes and inflammation [25]. Also, the results of De Servi and colleagues study showed that in patients with unstable angina, neutrophils, and monocytes showed a significantly higher expression of CD11b/CD18 adhesion receptors in coronary sinus than in aortic blood. The significant correlation between upregulation of CD11b/CD18 adhesion molecules on neutrophils and proximity of anginal episodes to blood sampling seems to support the hypothesis that neutrophil activation reflects the effects of recurrent ischemia on the myocardium rather than inflammatory activity in the damaged artery [26].

In addition to PNA, the interaction between platelets and monocytes in ACS underscores the intricate connection between inflammation and thrombosis in CVD. When a plaque ruptures, it triggers the activation of the inflammatory response, leading to an upsurge in TF expression and the initiation of extrinsic coagulation. Platelets, through PMA, stimulate the production of cytokines and TF in monocytes. Monocytes, in turn, become a significant source of microvesicle‐borne TF, promoting the accumulation of fibrin at the site of thrombus formation [11, 27].

Activated platelets form aggregates with monocytes, facilitated by P‐selectin. This interaction subsequently occurs with injured vascular endothelial and smooth muscle cells (SMCs), inducing the upregulation of matrix‐degrading enzymes. This upregulation is a crucial factor in the rupture of atheromatous plaques, leading to the breakdown of the fibrous cap [3].

The PMA can also induce the expression and secretion of monocyte chemoattractant protein‐1 (MCP‐1) and IL‐8 from monocytes in a P‐selectin/PSGL‐1‐dependent manner. Additionally, P‐selectin‐dependent interactions enhance TF expression, platelet‐activating factor (PAF) release, phagocytosis, and superoxide anion generation by monocytes. In collaboration with activated platelets, these factors contribute to the heightened instability of plaques, exacerbating the clinical symptoms of patients with ACS [3]. The activation and infiltration of platelets, along with monocytes/macrophages, play a crucial role in the initiation of ACS. Increased circulating PMAs have been reported in stable CAD, unstable angina, and acute MI [11].

In our study, we observed a significant elevation in the levels of PMAs in the ACS group compared to the healthy control group. Numerous studies have consistently demonstrated a substantial increase in PMA levels in patients with ACS compared to their healthy counterparts [3, 28, 29]. A growing body of evidence suggests that activated platelets induce a pro‐inflammatory monocyte phenotype, influencing the inflammatory response in both acute and CCS. This influence is mediated by direct interactions between platelets and monocytes, as well as the formation of PMA. This process results in the release of platelet‐derived cytokines, chemokines, and the shedding of procoagulant extracellular vesicles. Consequently, monocytes release inflammatory cytokines into circulation, trigger a procoagulant condition, and transform into proatherogenic macrophages [30]. The pro‐inflammatory cytokines are well‐known as other motivating factor in ACS development. The cytokines hold a pivotal role in both the pathogenesis and progression of CVDs.

Therefore, our study aimed to fill this gap by measuring the plasma levels of two key cytokines, namely, IL‐6 and TNF‐α. This investigation sought to provide valuable insights into the associations between these specific cytokines and the incidence and severity of ACS.

In our study, the concentration of IL‐6 in the group of patients with ACS significantly increased to four times higher than that in the healthy control group. As a member of the interleukin family, IL‐6 plays a pro‐inflammatory role, promoting the adhesion and aggregation of inflammatory cells throughout the organism. This finding underscores its involvement in the development of atherosclerosis and thrombosis. Elevated blood IL‐6 levels are associated with CVDs, including endothelial dysfunction, arterial stiffness, and atherosclerosis [31]. Furthermore, additional studies have indicated that IL‐6 is linked to adverse in‐hospital prognosis in ACS patients. It serves as an independent marker of increased mortality in unstable CAD patients, with the associated risk mitigated through dalteparin therapy [6, 32].

Numerous studies have shed light on the multifaceted involvement of IL‐6 and its diverse signaling pathways in various mechanisms contributing to both the formation and destabilization of atherosclerotic plaques. Upon release into the bloodstream, IL‐6 molecules activate inflammatory cells, leading to the release of chemokines and adhesion molecules, thereby triggering the aggregation of these cells to form atherosclerotic plaques. The subsequent release of ROS and the increased expression of metalloproteinases by activated inflammatory cells contribute to the instability of atherosclerotic plaques [22]. Moreover, IL‐6 can activate the complement system, influencing the synthesis of endothelin‐1 (ET‐1) and nitric oxide (NO), thereby altering endothelial function. Finally, IL‐6 may promote the expression of TF type I, plasminogen activator inhibition factor (PAI‐1), intensifying its activities and inducing coagulation abnormalities and thrombus formation [22]. In the context of CAD patients, elevated blood levels of IL‐6 may play a crucial role in the transformation of macrophages into foam cells during the atherosclerosis process [22].

The cytokine TNF‐α was also investigated in our study. The results demonstrated that the serum level of TNF‐α in patients with ACS was higher than that in healthy controls; however, this difference did not reach statistical significance. Interestingly, a study conducted by Diah and colleagues revealed no significant difference in TNF‐α levels among individuals with CAD, those with coronary slow flow (CSF), or healthy subjects [33]. Similarly, Popova and colleagues. conducted a study in which serum TNF‐α, IL‐6, and hs‐CRP levels at the 24th h were significantly elevated in both ACS patients with rheumatoid arthritis and ACS patients without rheumatoid arthritis, distinguishing them from controls with an accuracy ranging from 80% to 99%. Notably, by the 48th hour, the serum TNF‐α and IL‐6 levels in the ACS group without rheumatoid arthritis had decreased to levels comparable to those of the controls [34]. Conversely, Zare and colleagueset al., in their investigation measuring IL‐8 and TNF‐α levels before and after angiography, observed a significant reduction in IL‐8 levels postangiography, but TNF‐α levels did not decrease [35].

It is worth noting that despite inconsistencies in findings, several other articles have reported a higher level of TNF‐α in patients with ACS than in controls. The variations observed in these studies highlight the complexity of cytokine dynamics and the need for further research to elucidate the role of TNF‐α in the context of ACS [36]. One plausible explanation for the observed difference between IL‐6 and TNF‐α may lie in the distinct plasma half‐lives of these two cytokines. Other articles have noted that the half‐life of TNF‐α is considerably shorter than that of IL‐6 [37].

Multiple lines of evidence suggest the involvement of TNF‐related molecules in the development of ACS. The most compelling evidence pertains to the CD40L–CD40 interaction; however, several other members of the TNF superfamily, such as LIGHT, receptor activator of nuclear factor kappa‐Β ligand (RANKL), and TNF‐α, also appear to play a role in the immune‐mediated promotion of plaque instability. These pathways leading to plaque destabilization involve bidirectional interactions between platelets and endothelial cells/monocytes, activation of vascular smooth muscle cells (SMCs), and costimulatory effects on T cells. These processes collectively contribute to inflammation, thrombosis, matrix degradation, and apoptosis [38]. The identified TNF‐related pathways could contribute to the nonresolving inflammation characteristic of atherosclerotic disorders, representing pathogenic loops that operate during plaque rupture and the development of ACS. Importantly, these molecules present potential targets for therapeutic interventions in this disorder. Exploring these avenues could yield new and promising strategies for managing ACS [38, 39].

The outcomes of our study revealed a direct correlation between IL‐6 and PLAs (PMA and PNA) in the patient group, where both values increased. This finding aligns with observations made by other researchers in their respective studies.

Concerning the association between IL‐6 and PMA as well as PNA, various theories have been proposed. One line of thought is whether the secretion of IL‐6 triggers increased platelet aggregation with monocytes and neutrophils or whether platelet aggregation with leukocytes induces increased secretion of IL‐6. However, a bidirectional feedback relationship between these two parameters is evident.

Studies have shown that, first, inflammatory mediators such as the complement factors IL‐6, IL‐8, and TNF‐α, which are secreted from activated immune and vascular cells, contribute to platelet activation. They enhance adhesion to endothelial cells, increase collagen‐induced platelet aggregation, and reinforce the release of TXA2. This intricate interplay underscores the dynamic relationship between IL‐6 and the PLA, contributing to a deeper understanding of the complex mechanisms involved in cardiovascular pathophysiology [30]. Second, in the presence of platelets, plaque macrophages exhibit a pro‐inflammatory phenotype characterized by the upregulation of suppressor of cytokine signaling 3 (SOCS3). This upregulation, in turn, promoted the production of pro‐inflammatory cytokines, including IL‐6, IL‐1b, and TNF‐α. Third, the adhesion of monocytes to immobilized P‐selectin induces the secretion of various cytokines, such as TNF‐α, IL‐1, IL‐6, IL‐8, IL‐12, and macrophage inflammatory protein‐1 (MIP‐1).

In a study by Le Joncour and colleagues, an increase in the proportion of both PNA and PMA was observed in COVID‐19 patients compared to healthy donors. Additionally, a direct linear correlation between PNA and PMA levels and between PNA and IL‐6 was identified. This study further investigated the impact of sarilumab (an anti‐IL‐6 receptor) on PNA and PMA and revealed a significant decrease in PNA and PMA after treatment with sarilumab. These findings suggest that targeting the cytokine storm, particularly through the IL‐6 pathway, may reduce platelet/leukocyte complexes, potentially alleviating thrombotic/microthrombotic complications in patients with severe COVID‐19 [20].

In another study by Aberg and colleagues, an increased presence of PMA and PNA in patients with pulmonary hypertension, including its subgroups, was noted, with significant elevations compared to those in the control group. Additionally, the IL‐6 and TNF‐α levels were significantly higher in the patient group than in the control group. These findings underscore the potential link between PLAs and inflammatory cytokines in the context of pulmonary hypertension [40].

The findings from these studies, including our own, have consistently demonstrated a direct and linear relationship between IL‐6 levels and both PMA and PNA. This correlation implies a two‐way feedback relationship between IL‐6 and these aggregate levels, as evidenced by studies showing that blocking the IL‐6/IL6‐R/GP130 signaling complex leads to a reduction in PMA and PNA. Consequently, inhibiting IL‐6 has emerged as a potential therapeutic strategy to decrease PMA and PNA levels, thereby mitigating their role in the pathophysiology of various diseases [40, 41, 42].

ROC curve analysis was employed to determine which indicators could effectively differentiate the patient group from the healthy group. Our results indicated that PMA, PNA, and IL‐6 serve as robust markers for distinguishing patients from healthy individuals, whereas TNF‐α is a weaker marker for this purpose. These results, in addition to the good diagnostic power of these markers in ACS, show the relationship between them and the important role of these parameters in the pathogenesis of ACS, so the ROC curve shows that changes in these parameters can have prognostic value in addition to diagnostic value. These findings may also provide a new therapeutic strategy for the treatment of ACS [6, 43, 44, 45].

## 5 Conclusion

The results of our study showed that PMA, PNA, and IL‐6 are key elements in the pathogenesis of atherosclerosis as well as ACS and that these parameters can have diagnostic value. Additionally, a direct linear relationship between these parameters indicates a feedback effect, and there is a longitudinal relationship between them that can be a clue to providing a new treatment strategy for ACS. Inhibiting cytokine secretion and production prevented PLA formation by preventing the recruitment of more WBCs, especially monocytes and neutrophils, to atherosclerotic plaque sites. To achieve this goal, more studies are required in this field.

## Author Contributions

**Mohammad Ghorbani:** conceptualization, investigation, writing–review and editing, methodology. **Davood Bashash:** investigation, methodology. **Mohamad Esmail Gheydari:** investigation, methodology. **Mohammad Hossein Mohammadi:** investigation, methodology. **Hojat Shahraki:** investigation, methodology. **Somayeh Yazdanparast:** writing–review and editing, investigation. **Keyvan Olazadeh:** formal analysis, software. **Nazli Atashzar:** investigation. **Mohsen Hamidpour:** supervision, writing–review and editing, writing–original draft, project administration.

## Ethics Statement

Ethical approval was obtained from the Ethics Committee of Shahid Beheshti University of Medical Sciences under the approval code of IR.SBMU.RETECH.REC.1401.65. Additionally, written informed consent was obtained from all individuals. Written informed consent was obtained from all volunteers who participated in this study. The study was also carried out following relevant guidelines and regulations according to the Helsinki Declaration.

## Conflicts of Interest

The authors declare no conflicts of interest.

## Transparency Statement

The lead author Mohsen Hamidpour affirms that this manuscript is an honest, accurate, and transparent account of the study being reported; that no important aspects of the study have been omitted; and that any discrepancies from the study as planned (and, if relevant, registered) have been explained.

## Data Availability

The data supporting this study's findings are available on request from the corresponding author. The data are not publicly available due to privacy or ethical restrictions. Data are available from the corresponding author upon reasonable request.
